# Effects of *Eriobotrya japonica* (Thunb.) Lindl. Leaf Extract on Zebrafish Embryogenesis, Behavior, and Biochemical Pathways

**DOI:** 10.3390/molecules30153252

**Published:** 2025-08-03

**Authors:** Jorge Barros, Irene Gouvinhas, Carlos Venâncio, Daniel Granato, Ana Novo Barros, Luís Félix

**Affiliations:** 1 Centre for Research and Technology of Agro-Environmental and Biological Sciences (CITAB, Inov4Agro), University of Trás-os-Montes and Alto Douro (UTAD), Quinta de Prados, 5000-801 Vila Real, Portugal; igouvinhas@utad.pt (I.G.); cvenanci@utad.pt (C.V.); 2 Department of Agricultural Sciences, Higher Polytechnic Institute of Bengo, B. Caboxa|Dande, Bengo 244-2004, Angola; 3 Bioactivity & Applications Lab, Department of Biological Sciences, Faculty of Science and Engineering, School of Natural Sciences, University of Limerick, V94 T9PX Limerick, Ireland; granatod@gmail.com

**Keywords:** *Eriobotrya japonica* (loquat), *Danio rerio*, embryogenesis, behavior, biochemical pathways

## Abstract

*Eriobotrya japonica* (Thunb.) Lindl. leaves are rich in polyphenolic compounds, yet their toxicological effects in aquatic models remain poorly understood. This study evaluated the impact of a hydroethanolic *E. japonica* leaf extract on zebrafish embryos through the use of morphological, behavioral, and biochemical parameters. The 96 h LC_50_ was determined as 189.8 ± 4.5 mg/L, classifying the extract as practically non-toxic, according to OECD guidelines. Thereby, embryos were exposed for 90 h to 75 and 150 mg/L concentrations of the *E. japonica* leaf extract. While no significant effects were noted at the lowest concentration of 150 mg/L, significant developmental effects were observed, including reduced survival, delayed hatching, underdevelopment of the swim bladder, and retention of the yolk sac. These malformations were accompanied by marked behavioral impairments. Biochemical analysis revealed a concentration-dependent increase in superoxide dismutase (SOD) and catalase (CAT) activity, suggesting the activation of antioxidant defenses, despite no significant change in reactive oxygen species (ROS) levels. This indicates a potential compensatory redox response to a pro-oxidant signal. Additionally, the acetylcholinesterase (AChE) activity was significantly reduced at the highest concentration, which may have contributed to the observed neurobehavioral changes. While AChE inhibition is commonly associated with neurotoxicity, it is also a known therapeutic target in neurodegenerative diseases, suggesting concentration-dependent dual effects. In summary, the *E. japonica* leaf extract induced concentration-dependent developmental and behavioral effects in zebrafish embryos, while activating antioxidant responses without triggering oxidative damage. These findings highlight the extract’s potential bioactivity and underscore the need for further studies to explore its safety and therapeutic relevance.

## 1. Introduction

The search for natural products, such as plants for healing, is universal and as old as medicine itself. Its prominent role in traditional medicine systems dates back to Sumerian civilization [1]. *Eriobotrya japonica* (Thunb.) Lindl., commonly called loquat, is a fruit tree belonging to the *Rosaceae* family, with greater distribution in East Asia, especially in the Chinese subtropical region. The term ‘Thunb’ is short for Carl Peter Thunberg, the first botanist who described the species, but originally placed it in the genus *Mespilus* in 1780. The term ‘Lindl’, on the other hand, is the abbreviation of the name of botanist John Lindley, who was responsible for reclassifying the species into its current genus, *Eriobotrya*, in 1821 [2,3]. This plant possesses high medicinal value due to its various organs, which have been used for thousands of years in traditional medicine [4,5]. In China, the leaves are widely used in traditional herbal medicine for the treatment of coughs caused by lung inflammation, dyspnea due to asthma, hyperglycemia, cancer, fungal infections, and nausea associated with stomach upset, restlessness, and thirst. In Japan, the leaves are used in folk medicine for the treatment of stomach pain, ulcers, chronic bronchitis, cancer, and type 2 diabetes mellitus [6,7]. The extract of the leaves of *E. japonica* is rich in bioactive compounds, such as phenolics, which exhibit bioactivity capable of counteracting inflammation, diabetes, cancer, bacterial infection, aging, pain, allergies, and other health problems [4,8]. In a previous study conducted by our research group [9], the leaf extract of *E. japonica* from the Azores region was characterized in terms of its phytochemical composition and bioactivity. The extract demonstrated a high content of polyphenols and carotenoids, exhibiting significant antioxidant capacity, as determined by FRAP, DPPH, and ABTS assays. In addition, HPLC–DAD analysis enabled the identification and quantification of several phenolic compounds, including chlorogenic acid, neochlorogenic acid, caffeic acid, *p*-coumaric acid, quercetin derivatives (quercetin-3-*O*-rutinoside, quercetin-3-*O*-glucoside, quercetin-3-*O*-rhamnoside), and luteolin. These compounds, in clinical studies with humans and mice, were related to a hepatic anti-inflammatory response and a reduction in oxidative stress; anti-inflammatory action in regard to obesity-related gut microbiota inflammation in children; a reduction in oxidative stress and inflammation in high-fat diet (HFD)-induced diabetic rats through the modulation of AMPK/SIRT1/NF-κB/Nrf2 signaling pathways; a diet-induced reduction in high-density lipoprotein (HDL) hypercholesterolemic patients; restoration of the expression of inflammatory genes, including TNF-α, IL-1β, and IL-6, and antioxidant enzymes including SOD and GP_X_; a reduction in aortic lesions associated with atherosclerosis and the body weight of HFD-fed mice; a reduction in the levels of pro-inflammatory cytokines (TNF-α, IL-1β, IL-6) in the lungs and liver and further increased levels of anti-inflammatory cytokines (IL-4, IL-10); and the regulation of insulin, vitamins E and C, SOD, and GSH levels in the pancreas [10]. Thus, recent and modern pharmacological studies have shown that the leaves possess hypoglycemic, hepatoprotective, antitumor, antitussive, antiasthmatic, antibacterial, anti-inflammatory, antioxidant, dermatoprotective, cardioprotective, immunomodulatory, anti-osteoporotic, and muscle atrophy-relieving properties [7]. However, several studies, including those on the bioavailability, pharmacodynamics, pharmacokinetics, mechanism of action, and structure–activity relationships of phytoconstituents from *E. japonica* leaves, still lack comprehensive research [6].

Zebrafish (*Danio rerio*) is a teleost, known as a “bony”, tropical freshwater fish from the South Asian “Himalayan region” that lives in aquatic habitats, from slow-moving streams to stagnant rice paddies [11,12]. Scientific interest in zebrafish as an in vivo vertebrate model system for research in the areas of behavior, genetics, physiology, and biomedical science began in the 1960s, when molecular biologist George Streisinger studied its nervous system at the University of Oregon [12]. Since then, its scientific potential has been demonstrated in modelling human diseases and biological processes at various stages of drug development, as well as evaluating toxicity [13]. Studies involving zebrafish embryos and larvae provide extensive results on the pharmacological and toxicological effects in regard to high-throughput drug screening. Meanwhile the complex behavioral repertoire of zebrafish, their sensitivity to drugs, and the ability to respond to them in a similar way to humans support their usefulness for pharmacological and toxicological research [14]. Additionally, the use of zebrafish as a vertebrate model for scientific studies has some advantages over other mammalian models, namely because it is economical, it produces a large number (usually >100) of embryos from each mating session, the process involves external and rapid embryonic development [11,13], optical transparency occurs during the development period [14], it is easy to apply genetic manipulations, and approximately 70% of human genes have at least one apparent zebrafish ortholog [15,16]. Therefore, this model has been widely used in drug discovery for the identification of target and off-target effects, disease modelling, high-throughput screening of compounds for the inhibition or prevention of disease phenotypes, and the development of new drugs [13,17]. However, no studies were found in the literature that utilize this model to assess the effects of *E. japonica*, despite evidence indicating its immunostimulant properties during the early stages of carp culture [18] and the non-toxic effects of corosolic acid, a component isolated from *E. japonica* leaves, on zebrafish embryos [19].

Thus, this study aimed to investigate the effects of an E. japonica leaf extract on the embryogenesis, behavior, and biochemical pathways in zebrafish, with the goal of identifying potential toxic and therapeutic effects that may impact oxidative stress, inflammation, cytotoxicity, and neurotoxicity during the early stages of development.

## 2. Results

### 2.1. Effects of the Extract on the Survival Rate and Embryonic Development

When comparing developmental parameters over 96 h of exposure to 75 and 150 mg/L of E. japonica leaf extract and a negative control (Table 1), significant differences were found in the cumulative mortality. While the control group showed a steady mortality rate of around 16% with no major changes after 72 h, the group exposed to the highest concentration (150 mg/L) of leaf extract showed a sharp increase, reaching mortalities between 38% and 46% by 72 and 96 h, respectively. On the other hand, although exposure to 75 mg/L resulted in a slight increase in mortality, the difference was not statistically significant compared to the control group. At 24 hpf, embryonic development appeared normal, with no observable differences between the treatment groups. By 48 hpf, embryonic development remained normal, and the heart rate measurements showed no significant differences between the treatment groups. The hatching rate values, 94.1% for the 75 mg/L concentration group and 93.3% for the 150 mg/L concentration group, showed significant differences compared to the control group. Still, there were no significant differences between them. All of the other parameters evaluated showed no significant differences (*p* < 0.05, one-way ANOVA, followed by Tukey’s multiple comparison test).

**Figure 1:**
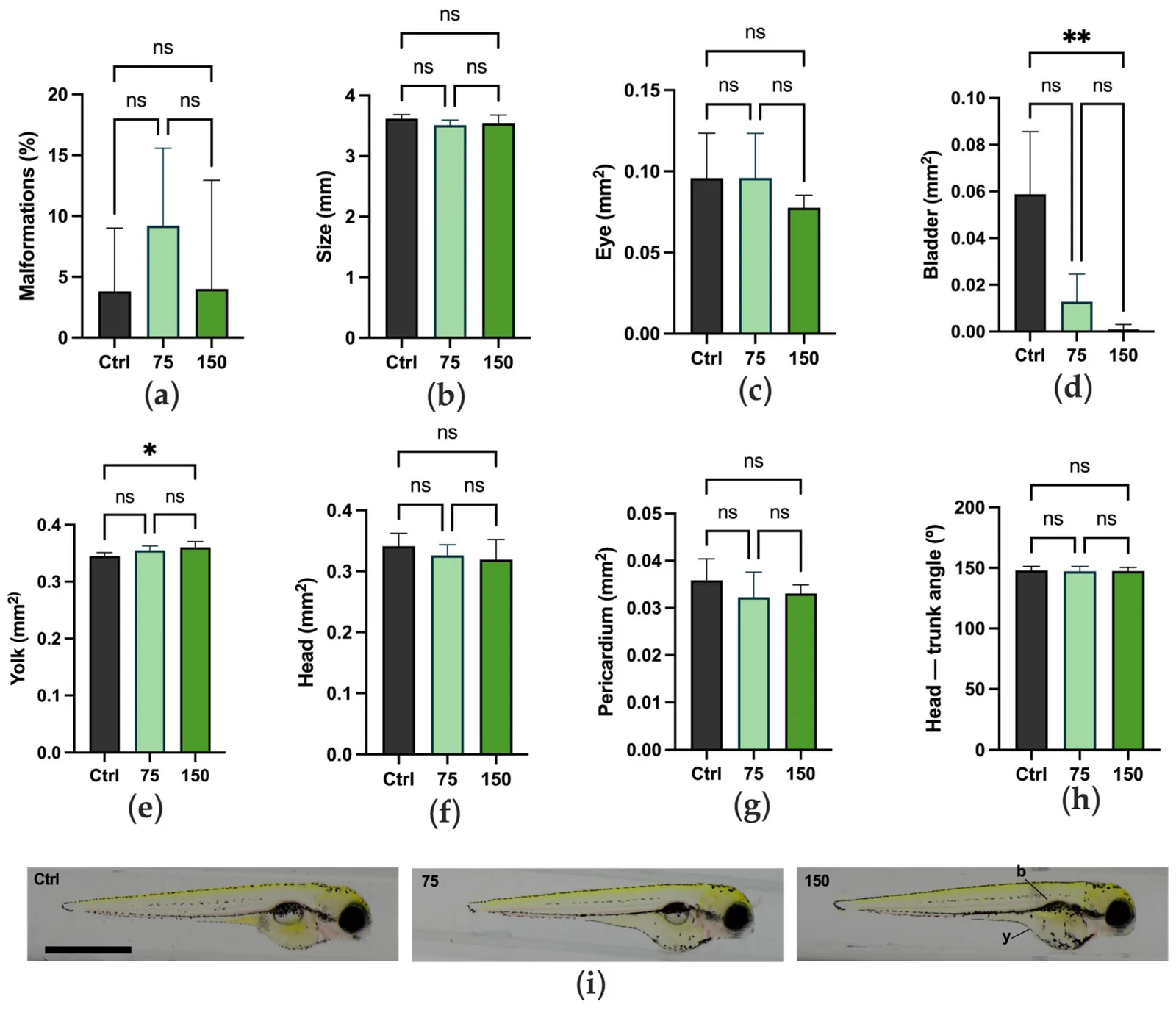
Morphological analysis of zebrafish embryos after 96 h of exposure to extract from *E. japonica* leaves. Results on the percentage of malformations (**a**), size (**b**), eye area (**c**), swim bladder area (**d**), yolk area (**e**), head area (**f**), pericardial area (**g**), head–trunk angle (**h**), and representative images of malformations observed in embryos (**i**). The values 75 and 150 denote concentrations in mg/L; * indicates significant differences (*p* < 0.05), ** indicates significant differences (*p* < 0.01), and ns indicates no significant differences between groups (*p* > 0.05); b: swim bladder, y: yolk sac.

Regarding the morphological analysis of zebrafish embryos after 96 h of exposure to the E. japonica leaf extract (Figure 1), no significant overall malformation rate was observed; however, an evaluation of individual parameters revealed notable changes. A concentration-dependent reduction in the swim bladder area was observed, with the highest concentration resulting in an almost complete absence of a visible swim bladder (*p* < 0.05, Kruskal–Wallis test, followed by Dunn’s test). An opposite trend was observed for the yolk area, with a higher area determined for the highest concentration (*p* < 0.05, Kruskal–Wallis test, followed by Dunn’s test). No other differences were observed for the remaining parameters after 96 h of exposure.

### 2.2. Impact on Locomotor Activity and Behavioral Responses

**Figure 2:**
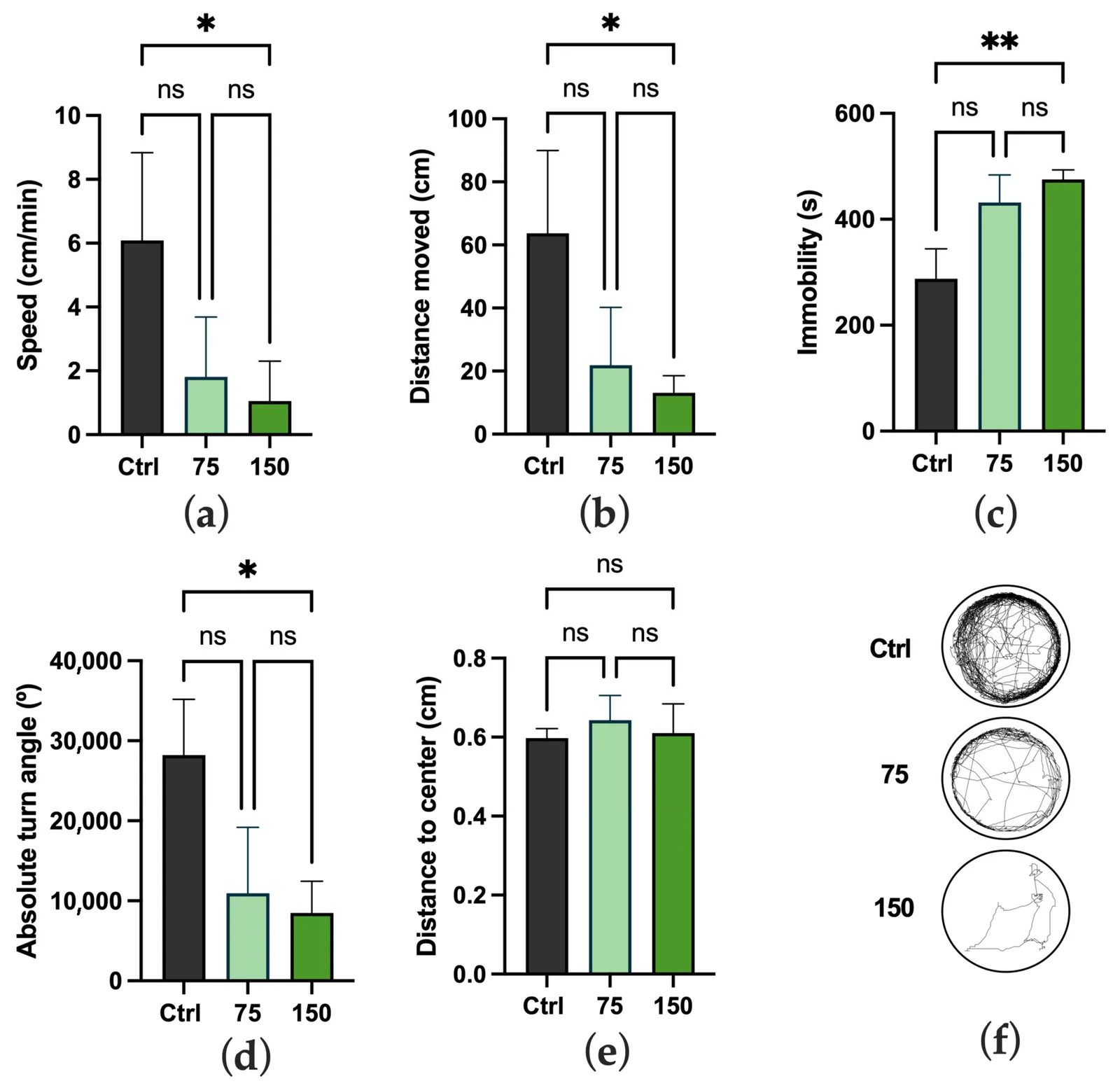
Determination of speed (**a**), distance moved (**b**), immobility (**c**), absolute turn angle (**d**), distance to center (**e**), and representative locomotion path of larvae exposed to the different *E. japonica* leaf extract concentrations (**f**). The values 75 and 150 denote concentrations in mg/L; * indicates significant differences (*p* < 0.05), ** indicates significant differences (*p* < 0.01), and ns indicates no significant differences between groups (*p* > 0.05).

The effects of the *E. japonica* leaf extract on the locomotor activity and behavior of the zebrafish embryos exposed to 75 and 150 mg/L (Figure 2) of the extract showed concentration-dependent variations. Significant differences were observed between the control group and the group exposed to the highest concentration (150 mg/L) in terms of speed (Figure 2a), distance moved (Figure 2b), immobility (Figure 2c), and absolute turning angle (Figure 2d) (*p* < 0.05, Kruskal–Wallis test, followed by Dunn’s test). No other difference was observed between the three treatment groups.

### 2.3. Biochemical Changes

**Figure 3:**
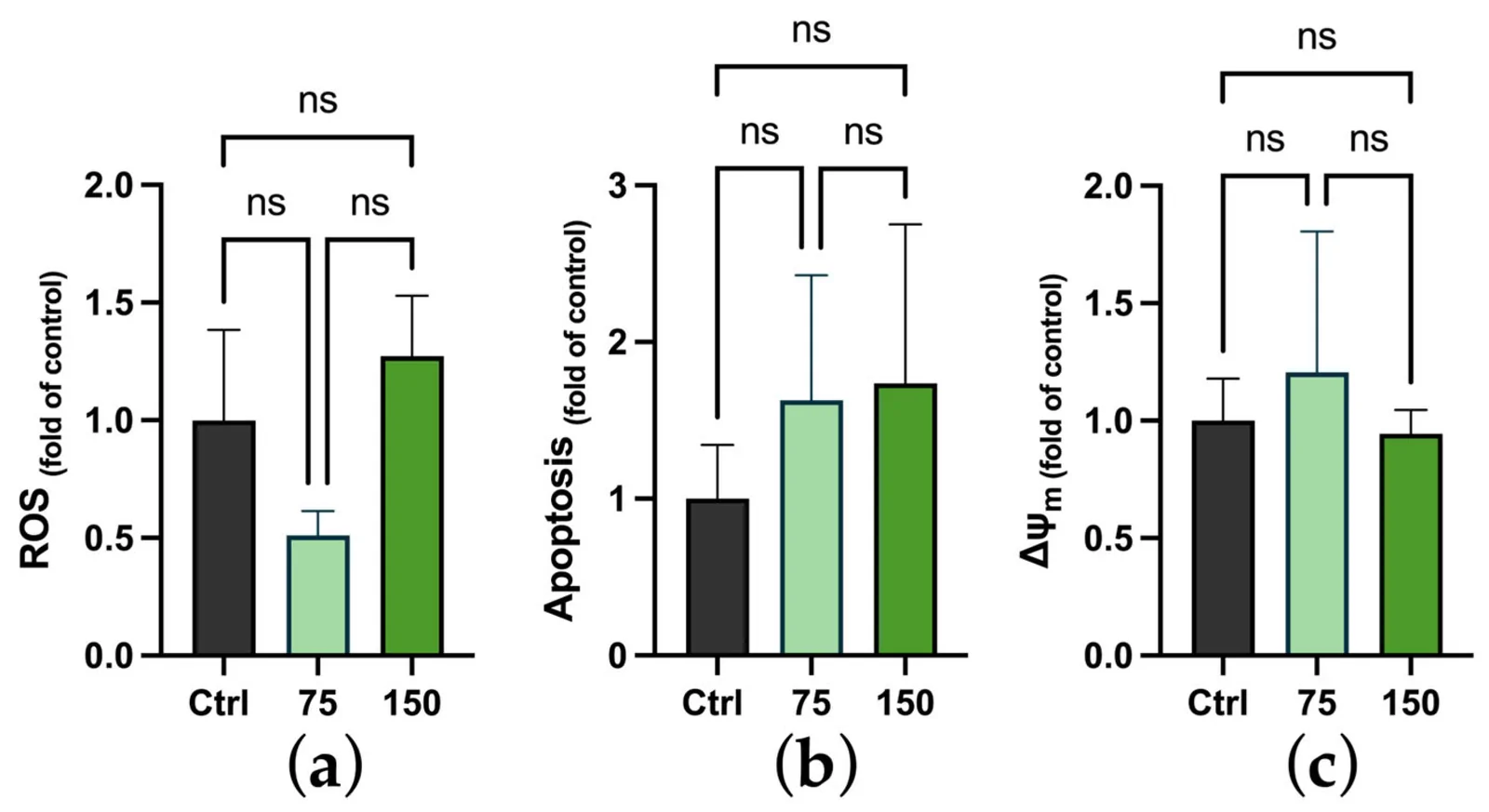
Levels of indicators of oxidative stress—ROS (**a**), cellular damage—apoptosis (**b**), and mitochondrial stress, neurotoxicity, and cellular dysfunction—ΔΨm (**c**). The values 75 and 150 denote concentrations in mg/L; ns indicates no significant differences between groups (*p* > 0.05).

**Figure 4:**
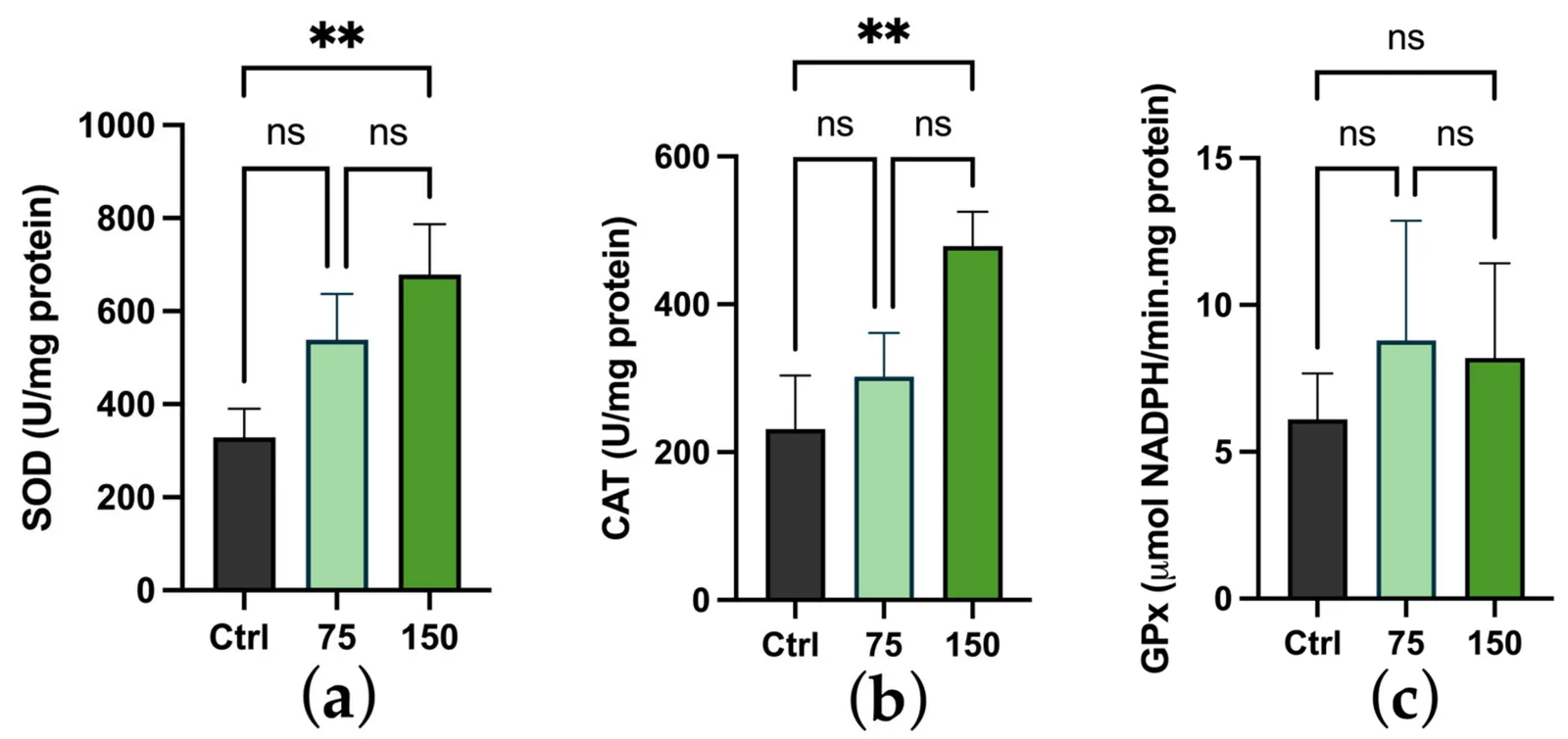
Biomarkers of the 1st line of antioxidant defense: SOD (**a**), CAT (**b**), and GPx (**c**) activity. Values are expressed as the mean ± standard deviation of 5 independent replicates. The values 75 and 150 denote concentrations in mg/L; ** indicates significant differences (*p* < 0.01), and ns indicates no significant differences between groups (*p* > 0.05).

**Figure 5:**
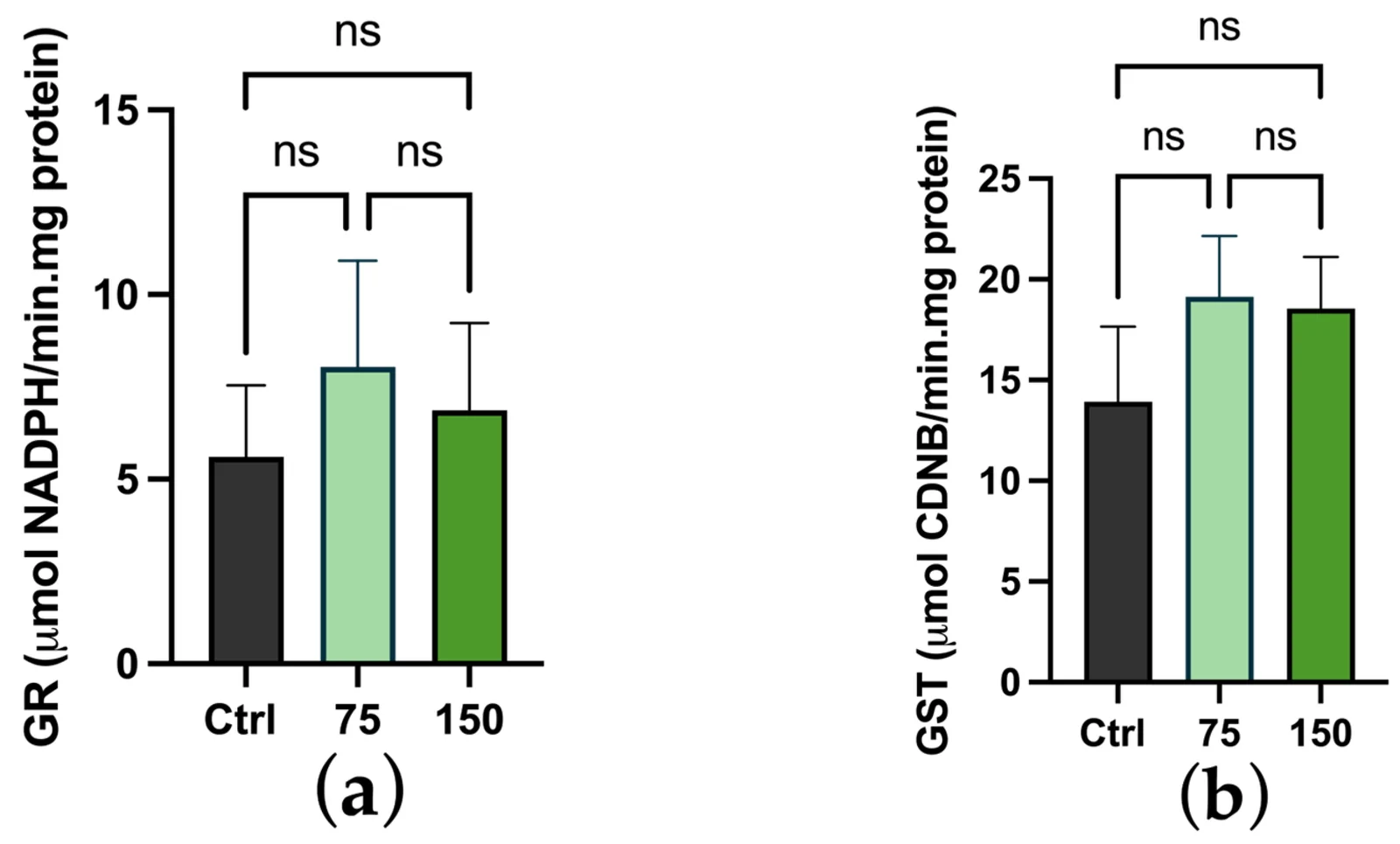
Metabolic biomarkers: GR (**a**) and GST (**b**) activity. The values 75 and 150 denote concentrations in mg/L; ns indicates no significant differences between groups (*p* > 0.05).

**Figure 6:**
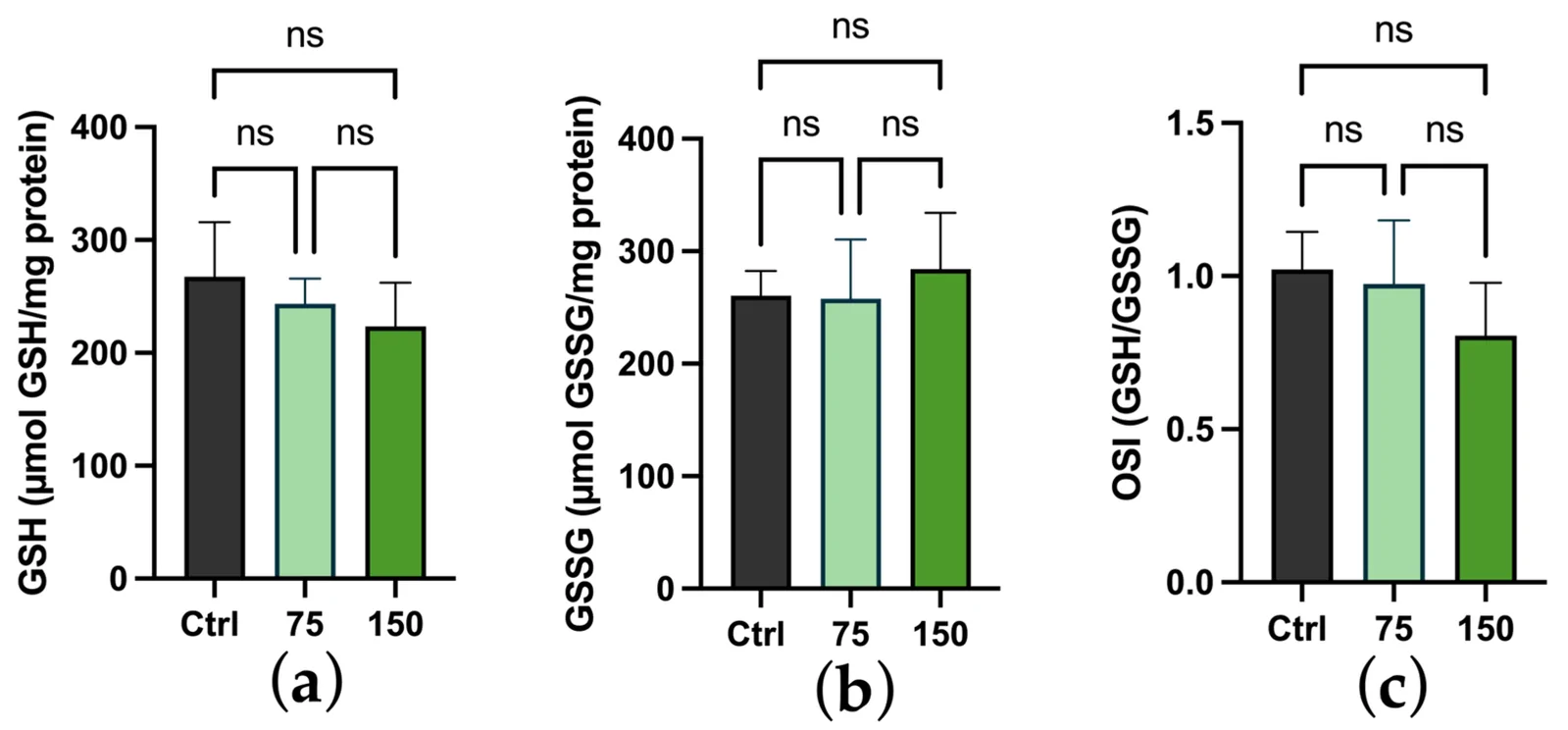
Levels of biomarkers of first-line antioxidant—GSH (**a**), metabolic—GSSG (**b**), and redox indices—OSI (**c**). The values 75 and 150 denote concentrations in mg/L; ns indicates no significant differences between groups (*p* > 0.05).

**Figure 7:**
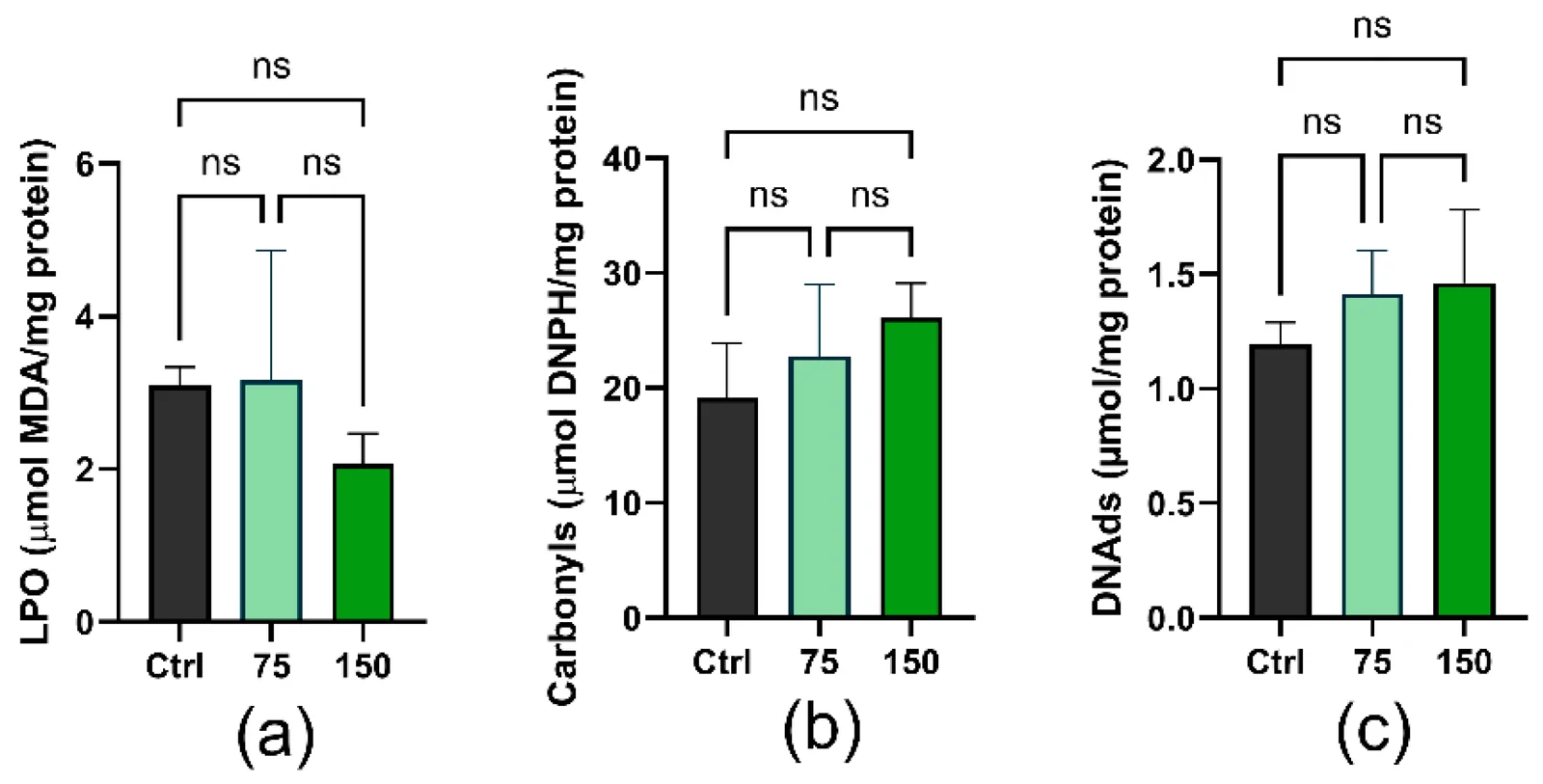
General indicators of cell damage and oxidative stress: LPO (**a**), protein carbonyls (**b**), and DNAds (**c**). The values 75 and 150 denote concentrations in mg/L; ns indicates no significant differences between groups (*p* > 0.05).

**Figure 8:**
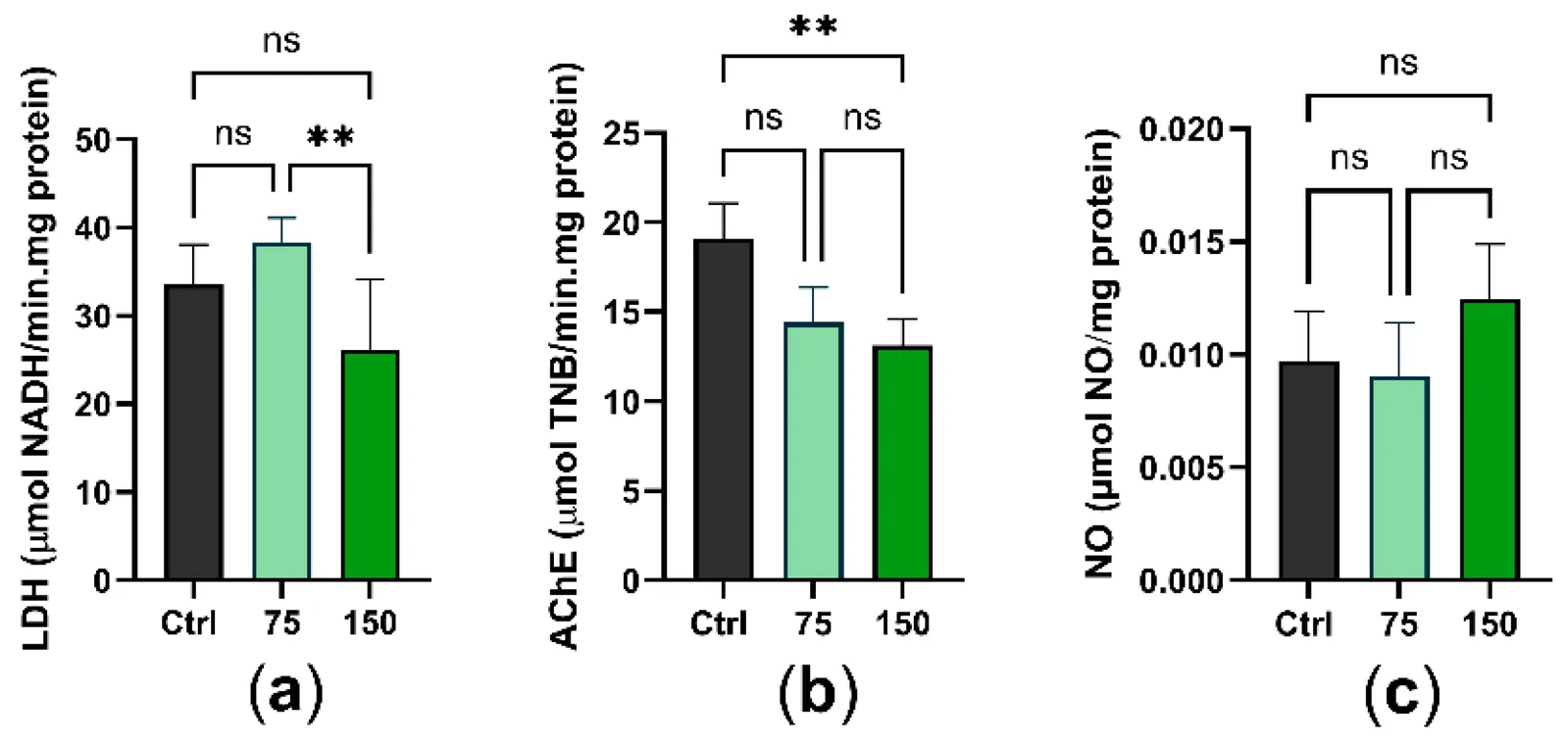
Levels of indicators of cell damage and damage to the nervous system (neurotoxicity): LDH activity (**a**), AChE activity (**b**), and NO levels (**c**). The values 75 and 150 denote concentrations in mg/L; ** indicates significant differences (*p* < 0.01), and ns indicates no significant differences between groups (*p* > 0.05).

Biochemical changes were evaluated through assessing different biomarkers. After 96 h of exposure to 75 and 150 mg/L of E. japonica leaf extract, the levels of ROS, apoptosis, and mitochondrial membrane potential (Figure 3) showed no significant differences compared to the control group (*p* < 0.05). In regard to the analysis of first-line antioxidant defense biomarkers (Figure 4), including SOD, CAT, and GPx, significant differences in the SOD activity were observed between the control group and the group exposed to the highest concentration (150 mg/L) of leaf extract. A similar pattern was observed for CAT activity, while no significant differences were recorded between the groups for the GPx activity. The glutathione-dependent biomarkers (GR and GST) showed no significant differences at either concentration compared to the control group (Figure 5a,b). Similarly, glutathione levels (GSH and GSSG) remained unchanged by the treatment (Figure 6), with no resulting alterations in the oxidative stress index (OSI). Regarding oxidative damage, assessed through lipid peroxidation, protein carbonylation, and DNA double-strand breaks (Figure 7), no significant differences were observed between the groups (*p* < 0.05). In regard to metabolism, neurotransmission, and inflammation biomarkers (Figure 8a,b,c), a significant decrease in AChE activity was observed at the highest concentration tested, compared to the control group (*p* < 0.05). Additionally, differences in the LDH activity were noted between the groups exposed to the 75 and 150 mg/L concentrations. However, no differences were observed in the NO levels.

## 3. Discussion

Despite some studies describing the biological effects of *E. japonica* compounds and extracts on aquatic animals [18,19], no research has specifically investigated the toxicological outcomes of *E. japonica* exposure using zebrafish as a model organism. Therefore, this study aimed to evaluate the effects of an ethanolic extract of *E. Japonica* leaves on zebrafish embryos, based on morphological, behavioral, and biochemical parameters. The results showed significant effects at 150 mg/L, including increased mortality, reduced locomotor activity, and an increase in the activity of SOD and CAT, while decreasing the AChE activity.

The initial phase of this study involved determining the LC_50_ of the prepared plant extract, using the zebrafish embryo toxicity assay. As no prior toxicological assessments of this specific extract were found in the existing literature, this investigation provides novel insights into its safety profile. The experimentally determined LC_50_ value of 189.8 ± 4.5 mg/L places the extract within the practically non-toxic category for aquatic organisms, as defined by the OECD guidelines [20]. Comparative data from the literature indicate a range of LC_50_ values for ethanolic plant extracts, some reporting similar, higher, or lower toxicity thresholds depending on the plant species, extraction methods, and experimental conditions [21]. Therefore, given the relatively low acute toxicity observed and the lack of available literature, the next step aimed to explore the sublethal and developmental effects of this extract at lower concentrations, which are critical for elucidating its pharmacological potential and ensuring its safety for prospective therapeutic applications.

In this study, the hydroethanolic extract of *E. japonica* was shown to exert stage-specific, time-dependent, and concentration-dependent effects on the development of zebrafish embryos. Notably, the embryonic survival rates significantly decreased at the highest concentration tested, which was expected given its proximity to the established LC_50_ threshold, indicating a potential toxicological boundary for the extract. Additionally, delayed hatching was observed across both tested concentrations, suggesting a broader impact on embryogenesis, beyond acute toxicity. Furthermore, morphological assessments revealed significant developmental abnormalities at the highest tested concentration of the extract (150 mg/L), including underdevelopment of the swim bladder and an enlarged yolk sac. These phenotypic disruptions are commonly associated with impaired organogenesis and metabolic dysregulation during embryonic development. Notably, the leaves of *E. japonica* are a well-documented source of bioactive polyphenols, particularly hydroxycinnamic acids (such as chlorogenic and neochlorogenic acid), flavan-3-ols, flavonols, and flavones [10]. For instance, in the previous study carried out by our research group [9], *E. japonica* leaf extracts showed 111.58 ± 6.08 mg GA/g DW of total phenols, 137.00 ± 1.11 mg GA/g DW of *ortho*-diphenols, and 54.99 ± 4.84 mg CAT/g DW of flavonoids. In addition, the identification and quantification process carried out using HPLC–DAD showed remarkably high levels of neochlorogenic acid (15.33 ± 0.34 mg/100 g DW) and chlorogenic acid (22.21 ± 0.60 mg/100 g DW), compared to the other compounds. These polyphenolic compounds are known to modulate developmental signalling pathways in aquatic organisms. Among these compounds, chlorogenic acid has been specifically reported to influence zebrafish embryonic mortality and hatchability at low concentrations. In contrast, higher concentrations can lead to swim bladder malformation or loss, as well as delayed yolk sac absorption in zebrafish embryo [22]. Given that the estimated concentration of chlorogenic acid in the higher extract concentration used in the present study may exceed this threshold, it is plausible that this compound significantly contributed to the observed developmental delays and reduced survival rates.

In addition to the morphological abnormalities, behavioral analyses revealed significant impairments in larval locomotion at the highest concentration of the extract (150 mg/L), including decreased swimming speed and distance travelled, increased immobility, and a reduced turn angle. These behavioral deficits may be closely linked to the observed morphological malformations, specifically, swim bladder underdevelopment and yolk sac retention. In fact, the swim bladder plays a critical role in buoyancy regulation [23,24], and its underdevelopment can lead to significant impairments in locomotion and stability. Similarly, an enlarged yolk sac often reflects delayed nutrient absorption and insufficient energy mobilization [25], which may further compromise overall motility [26]. However, among these factors, the failure of swim bladder inflation is likely the more critical contributor to the observed locomotor deficits, given its direct role in facilitating active swimming and vertical positioning in the water column. This trend reinforces the idea that concentrations of *E. japonica* leaf extract approaching the LC_50_ threshold elicit behavioral responses compatible with mild-to-moderate neurotoxicity, while lower concentrations do not functionally compromise the nervous system of embryos. In this context, although no study has directly evaluated the behavioral toxicity of *E. japonica* extracts in zebrafish, the known bioactive compounds in its phytochemical profile, particularly chlorogenic acid, which was significantly distinguished from the other compounds in the previous study by our research group, offer plausible mechanistic explanations for the observed effects. However, further studies are needed to clarify this hypothesis. In this context, the development of the swim bladder involves specific pathways [23], and chlorogenic acid has been shown to modulate different signaling pathways [27,28] that warrant further investigation.

In particular, the impaired swim bladder has been linked to oxidative stress-induced disruption of critical developmental signalling pathways, notably the Wnt and Hedgehog pathways [29,30]. At the same time, chlorogenic acid supplementation has been shown to have a potential protective role against oxidative damage during embryogenesis [31]. Given these conflicting mechanistic insights, several oxidative stress markers were evaluated following exposure to the *E. japonica* leaf extract to better understand the potential underlying causes of the observed morphological and behavioral alterations. The results demonstrated a concentration-dependent increase in the activity of the antioxidants, SOD and CAT, which are well-established biomarkers of the oxidative stress response [32]. This increase in enzyme activity is commonly observed as a cellular adaptive mechanism to combat oxidative damage induced by environmental stressors. However, despite this upregulation of antioxidant defenses, ROS production and markers of oxidative damage remained consistent across the treatments, suggesting that the embryonic cells recognized a pro-oxidant signal induced by the extract and responded by activating their endogenous antioxidant systems as a compensatory mechanism, thus maintaining overall redox homeostasis. Similar patterns have been reported in other studies involving plant extracts and zebrafish at different developmental stages, wherein the increase in antioxidant defenses did not result in a concomitant rise in oxidative damage [33,34,35]. Overall, these findings highlight the ability of the *E. japonica* leaf extract to elicit a balanced response that promotes cellular homeostasis. In this context, its composition, based on chlorogenic acid, may have activated signaling cascades via the Nrf2 pathway or other redox regulatory pathways [36,37], which warrant further investigation. In addition to modulating oxidative stress markers, exposure to the highest concentration of the extract resulted in an inhibition of AChE activity. AChE is a critical enzyme in the cholinergic system, responsible for the hydrolysis of the neurotransmitter acetylcholine at synaptic clefts, thereby terminating neural transmission and preventing excessive neuronal excitation [36]. The inhibition of AChE disrupts this balance, leading to the prolonged stimulation of cholinergic receptors, which can manifest as neurotoxic symptoms, including altered behavior, motor impairment, and neuromuscular dysfunction [37]. In this context, many naturally occurring compounds from plants have shown AChE inhibitory activity [38], which may help explain the concurrent behavioral abnormalities observed [36]. In contrast, AChE inhibition is often associated with neurotoxicity; it also forms the basis of therapeutic strategies for neurodegenerative disorders, such as Alzheimer’s and Parkinson’s disease [38,39]. Thus, the observed AChE-inhibitory activity may not be an exclusive indicator of neurobehavioral toxicity. Still, it could also point towards potential therapeutic relevance. Notably, a non-significant decrease in AChE activity was also detected at the lowest concentration (75 mg/L) of the extract, further supporting the possibility of a modulatory rather than an overtly toxic effect at submaximal doses.

## 4. Material and Methods

### 4.1. Collection of the Leaves of E. japonica and Preparation of the Extract

The leaves of *E. japonica* were harvested at Quinta de São Gonçalo, São Miguel Island, Azores Region, specifically at the Bio Kairós cooperative, located at the geographical coordinates, 37.7480° N, 25.6575° W, at the end of 2024. Subsequently, the leaves were transported in vacuum packs to the laboratory and frozen at −80 °C. The leaves were then ground into a powder using a Moulinex knife mill, standardized with a Tyler mesh sieve, and dissolved in an ethanol/water (70:30 *v*/*v*) solution at a concentration of 8 mg/mL. The hydroethanolic extract was evaporated using a Lab Tech rotary evaporator, then frozen at −80 °C, and subsequently lyophilized. The final yield was 56.8%.

### 4.2. Maintenance of Zebrafish

The embryos used were collected from the breeding tanks of adult Danio rerio fish, resulting from spawning, in the Ecotoxicology vivarium at the University of Trás-os-Montes and Alto Douro (Vila Real, Portugal). The adult fish were kept under the conditions described before [39], namely involving aerated and filtered tap water, with a pH of 7.3–7.5 and a temperature of 28 ± 0.5 °C. In terms of diet, the fish were fed twice a day on a nutritionally balanced and standardized diet for zebrafish (Zebrafeed, Sparos Lda., Quelfes, Portugal), according to previous studies [40,41]. Their reproduction resulted from mating between females and males (a ratio of 1:2), which occurred at night in glass tanks. Spawning was carried out at 8 a.m. under light induction. The eggs were collected at 9 a.m. and submitted to a bleaching process by washing the eggs with a 0.5% chloramine-T solution prepared in E3 buffer (NaCl 5 mM, KCl 0.17 mM, CaCl_2_ 0.33 mM, and MgSO_4_ 0.33 mM) (pH 7.2), followed by two washes in E3 buffer [42]. The experimental tests were carried out in adherence to animal welfare standards [43], the European Directive No. 2010/63/EU, and Portuguese legislation, in particular (Decree-Law 113/2013) [44].

### 4.3. Exposure to the Extract

Initially, experimental studies were conducted to determine the lethal concentration that causes 50% mortality (LC_50_) in zebrafish embryos, in accordance with the OECD Test Guideline 236 and the method previously described [45]. In this case, embryos were collected in the early stage of blastula (~2.0 h after fertilization (hpf)), after washing, and distributed in 6-well plates of 5 mL, with 20 random embryos placed into each well (n = 1). More than 5 independent replicates were exposed to different freshly prepared concentrations of E. japonica leaf extract (25, 50, 100, 200, and 400 mg/L), with E3 buffer as the negative control. Solution replacement and mortality recording were performed at 8, 24, 48, 72, and 96 hpf. After 96 h of exposure, the LC_50_ was calculated, yielding a value of 189.8 ± 4.5 mg/L (Figure S1). This value was obtained using the Abbott formula (1925) and probit analysis (Finney, 1971 [46]). Based on this previously determined LC_50_ value, concentrations of 75 and 150 mg/L were defined for the subsequent exposure experiments. To determine the effects of the extract on embryogenesis, behavior, and biochemical pathways, the embryos (~2.0 h after fertilization (hpf)) were distributed into 6-well plates of 5 mL, with 50 random embryos placed into each well (n = 1), and more than 5 independent replicates were exposed to the E3 medium, 75 or 150 mg/L of the extract. Solution replacement, mortality recording, removal of dead eggs, and other observable and specific outcomes (developmental, behavioral, and biochemical parameters) described below, were performed at 8, 24, 48, 72, and 96 hpf [41,47].

### 4.4. Embryotoxicity

Mortality was assessed at 8, 24, 48, 72, and 96 hpf and the dead animals were removed. Tail and head detachment, somite and eye development were observed at 24 hpf. Spontaneous movements were also counted over a period of 20 s, in 10 randomly selected animals from each group. The formation of pigmentation and the presence of edema (cardiac and yolk sac) were observed at 48 hpf. Heartbeats were counted for 15 s in 10 randomly selected animals from each group. The response to touch, the hatching rate, and malformations were analyzed at 72 hpf. At 96 hpf, the malformations (size, angle between head and tail, and areas of the eye, head, yolk sac, and pericardial space) of 10 animals randomly removed from each group were evaluated (n = 1) by immobilizing the larvae inside a microcapillary tube containing 1% of methylcellulose solution. The analysis was conducted using a stereomicroscope Olympus SZX7 (Tokyo, Japan), coupled to a digital camera, Olympus EP50 (Tokyo, Japan). A digital image analysis software (ImageJ Version 1.54p) was used for the measurements [41].

### 4.5. Behavior

At the end of the exposure period, 8 non-malformed larvae from each exposure group were analyzed in a 12-well flat-bottom plate containing an agarose ring, and with the use of an LCD computer screen to present a white PowerPoint presentation. After a 5 min habituation period, the well was filmed from above for 10 min using a mobile phone (1920 × 1080/30 fps). The total distance traveled, immobility time, and mean velocity were assessed to the measure activity, while the mean distance to the center of the well was indicative of anxiety behavior, specifically thigmotaxis. All these parameters were analyzed using the video tracking software, ANY-Maze (version 7.08, Stoelting Co., Wood Dale, IL, USA) [48].

### 4.6. Multiparameter Detection

At the end of all the trials, 10 animals (n = 1) were selected from each replicate for the quantification of the ROS, apoptosis, and mitochondrial membrane potential (ΔΨm), as described previously. In summary, the ROS levels were quantified after soaking the animals in a dichlorodihydrofluorescein diacetate solution (DCFH-DA, 20 μg/mL) for 1 h. The ΔΨm was determined using a fluorescent probe (JC-1, 5.0 μM) for 30 min, while apoptosis was assessed using acridine orange (AO, 10 μg mL^−1^) for 15 min. After exposure to each probe, 96 hpf larvae were washed, collected in 200 μL of E3 buffer and stored at −20 °C until homogenized in a Biobase BHY-1 (Jinan, China) and centrifuged at 12,000× *g* for 10 min at 4 °C. Supernatants were collected and fluorescence measured in excitation/emission of 485/535, 485/535, and 485/590, and 485/535 nm for the ROS, ΔΨm, and apoptosis, respectively, using a Cary Eclipse fluorescence spectrophotometer (Varian, Palo Alto, CA, USA) [41].

### 4.7. Biochemical Analysis

At 96 hpf, the surviving embryos from each replicate (around 30, n = 1) were collected and homogenized in cold HEPES buffer (0.32 mM sucrose, 20 mM HEPES, 1 mM MgCl_2_, and 0.5 mM phenylmethylsulfonyl fluoride [PMSF], pH 7.4) in a Biobase BHY-1 (Jinan, China). Following centrifugation at 12,000× *g* for 10 min at 4 °C, the protein of the supernatant was determined by measuring the absorbance at 280 nm, using a Take3 plate (BioTek Instruments, Winooski, VT, USA). The different enzymatic activities and oxidative stress markers were measured using either a PowerWave XS2 microplate scanning spectrophotometer (BioTek Instruments, Winooski, VT, USA) or a Cary Eclipse fluorescence spectrophotometer (Varian, Palo Alto, CA, USA) at 30 °C, as described before. The activity of superoxide dismutase (SOD) was evaluated according to its ability to inhibit the photochemical reduction of nitroblue tetrazolium (NBT) at 560 nm. Catalase (CAT) activity was determined by monitoring the reduction in absorbance of a hydrogen peroxide solution at 240 nm. The activities of glutathione reductase (GR) and glutathione peroxidase (GPx) were measured at 340 nm using the extinction coefficient of NADPH. Glutathione-S-transferase (GST) activity was quantified at 340 nm, based on the formation of a conjugate between reduced glutathione (GSH) and 1,2-chloro-2,4-dinitrobenzene (CDNB). The levels of GSH and oxidized glutathione (GSSG) were assessed with excitation at 320 nm and emission at 420 nm, and the GSH/GSSG ratio was used to calculate the oxidative stress index (OSI). Lipid peroxidation was measured at 530 nm via the reaction of malondialdehyde (MDA) with thiobarbituric acid (TBA). Protein carbonylation was determined at 450 nm, using the DNPH extinction coefficient. Lactate dehydrogenase (LDH) activity was assessed at 340 nm through the sodium pyruvate-mediated oxidation of NADH. Acetylcholinesterase (AChE) activity was quantified at 405 nm. DNA strand breaks were measured using excitation at 360 nm and emission at 450 nm [48].

### 4.8. Statistical Analyses

The data were evaluated for normality using the Shapiro–Wilk test and for homogeneity of variances, using the Brown–Forsythe test. Parametric data, expressed as the mean ± standard deviation, were analyzed using a one-way ANOVA, followed by Tukey’s post hoc multiple comparisons test. Nonparametric data, displayed as the median with the interquartile range, were analyzed using the Kruskal–Wallis test, combined with Dunnett’s multiple comparisons test. Statistical analyses and graphical representation were performed using GraphPad Prism software version 10 for Windows (GraphPad, Inc., San Diego, CA, USA), with statistical significance set at a two-tailed *p*-value < 0.05.

## 5. Conclusions

Overall, this study demonstrates that an *E. japonica* leaf extract induces concentration-dependent behavioral and developmental changes in zebrafish embryos, while modulating enzymatic antioxidant defenses, without provoking oxidative damage or cellular damage. Although no oxidative damage was detected, the inhibition of AChE and developmental changes at higher concentrations highlight the importance of dose when considering safety and potential therapeutic applications. These effects can be partially attributed to bioactive compounds, such as chlorogenic acid, which is known to modulate redox-sensitive and developmental signaling pathways (e.g., Nrf2, Wnt, and Hedgehog). Thus, future studies should focus on unraveling the molecular mechanisms underlying these effects, investigating long-term and multigenerational impacts, and evaluating the therapeutic potential of individual phytoconstituents on neuroprotection, redox homeostasis, and their relevance to human health.

## Figures and Tables

**Table 1:** Developmental parameters evaluated throughout the 96 h of exposure of zebrafish embryos to the 75 and 150 mg/L of an *Eriobotrya japonica* (Thunb.) Lindl. leaf extract.

Time (hpf)		Groups (mg/L)			Statistical Test	*p*-Value
	Parameters	Control	75	150		
8	Mortality (%)	4.0 (1.0–6.0)	2.0 (2.0–5.0)	2.0 (1.0–5.0)	X^2^ (2) = 0.63	0.756
24	Cumulative mortality (%)	15.2 ± 4.2	17.2 ± 2.3	13.6 ± 3.3	F (2, 12) = 1.47	0.269
	Non-detected tail (%) ^1^	ND	ND	ND	NA	NA
	Non-detected head (%) ^1^	ND	ND	ND	NA	NA
	Non-detected somites (%) ^1^	ND	ND	ND	NA	NA
	Spontaneous movements (mpm)	5.8 ± 1.0	6.2 ± 1.6	5.7 ± 1.6	F (2, 12) = 0.19	0.829
48	Cumulative mortality (%)	15.2 ± 4.2	18.4 ± 3.8	16.4 ± 3.3	F (2, 12) = 0.92	0.426
	Eye not developed (%) ^1^	ND	ND	ND	NA	NA
	Otolith not developed (%) ^1^	ND	ND	ND	NA	NA
	Absent blood circulation (%) ^1^	ND	ND	ND	NA	NA
	Non-visible pigmentation (%) ^1^	ND	ND	ND	NA	NA
	Heartbeat (bpm)	123.2 ± 9.5	116.6 ± 2.2	118.2 ± 2.9	F (2, 12) = 1.72	0.220
72	Cumulative mortality (%)	16.0 (11.0–19.0) ^a^	32.0 (26.0–32.0) ^ab^	38.0 (34.0–44.0) ^b^	X^2^ (2) = 12.64	<0.0001
	Hatching rate (%)	100 (97.6–100) ^a^	94.1 (93.0–95.8) ^b^	93.3 (83.9–96.8) ^b^	X^2^ (2) = 9.20	0.002
	Oedema presence (%) ^1,2^	ND	ND	ND	NA	NA
	Touch response (%)	100 (100–100)	100 (95.0–100)	100 (95.0–100)	X^2^ (2) = 1.08	>0.999
96	Cumulative mortality (%)	16.0 (11.0–19.0) ^a^	32.0 (26.0–34.0) ^ab^	46.0 (43.0–50.0) ^b^	X^2^ (2) = 12.57	<0.0001

^1^ Parameter quantified as present/absent. ^2^ Yolk sac and oedema were quantified as being only one. The analysis was performed with 5 independent replicates, each containing 10 randomly selected animals, and the results are expressed as the median and interquartile ranges. The data were statistically analyzed using a one-way ANOVA, followed by Tukey’s multiple comparison test, or the Kruskal–Wallis test, followed by Dunn’s test. Different lowercase letters indicate significant differences between groups (*p* < 0.05) within the same row; mpm: movements per minute; bpm: beats per minute; ND: non-detected; NA: not available.

## Data Availability

Data is contained within the article and Supplementary Materials.

## Supplementary Materials

The following supporting information can be downloaded at: https://www.mdpi.com/article/10.3390/molecules30153252/s1, Figure S1: LC_50_.

## Abbreviations

AChE (Acetylcholinesterase); ABTS (2,2′-Azino-bis (3-ethylbenzothiazoline-6-sulfonic acid)); AMPK (AMP-Activated Protein Kinase); CAT (Catalase); CDNB (1,2-chloro-2,4-dinitrobenzene); DPPH (2,2-Diphenyl-1-picrylhydrazyl); DNA (Deoxyribonucleic Acid); FRAP (Ferric-reducing antioxidant power); GPx (glutathione peroxidase); GR (glutathione reductase); GSH (reduced glutathione); GSSG (oxidized glutathione); GST (Glutathione-S-transferase); HFD (high-fat diet); HPLC–DAD (high-performance liquid chromatography with diode-array detection); IL-1β (interleukin 1β); IL-6 (interleukin six); LC_50_ (lethal concentration that causes 50% mortality); LDH (Lactate dehydrogenase); LPO (Lipid Peroxidation); MDA (malondialdehyde); ΔΨm (mitochondrial membrane potential); NBT (nitroblue tetrazolium); NF-kB (nuclear factor kappa B); NO (nitric oxide); Nrf2 (Nuclear factor erythroid 2); OECD (Organisation for Economic Co-operation and Development); OSI (oxidative stress index); PMSF (phenylmethylsulfonyl fluoride); ROS (reactive oxygen species); SIRT1 (Sirtuin 1); SOD (superoxide dismutase); T2DM (2 diabetes); TBA (thiobarbituric acid); TNF-α (tumor necrosis factor-alpha).
