# Correction: Barros et al. Effects of *Eriobotrya japonica* (Thunb.) Lindl. Leaf Extract on Zebrafish Embryogenesis, Behavior, and Biochemical Pathways. *Molecules* 2025, *30*, 3252

**DOI:** 10.3390/molecules31162739

**Published:** 2026-08-07

**Authors:** Jorge Barros, Irene Gouvinhas, Carlos Venâncio, Daniel Granato, Ana Novo Barros, Luís Félix

**Affiliations:** 1 Centre for Research and Technology of Agro-Environmental and Biological Sciences (CITAB, Inov4Agro), University of Trás-os-Montes and Alto Douro (UTAD), Quinta de Prados, 5000-801 Vila Real, Portugal; igouvinhas@utad.pt (I.G.); cvenanci@utad.pt (C.V.); 2 Department of Agricultural Sciences, Higher Polytechnic Institute of Bengo, B. Caboxa|Dande, Bengo 244-2004, Angola; 3 Bioactivity & Applications Lab, Department of Biological Sciences, Faculty of Science and Engineering, School of Natural Sciences, University of Limerick, V94 T9PX Limerick, Ireland; granatod@gmail.com

**Error in Figure 7**

In the original publication [1], there was a mistake in Figure 7 as published. The y-axis for DNA damage was incorrectly expressed as “μmol Pi/mg protein” when it should be “DNAds (μmol/mg protein)”. In the same figure, carbonyls should not be reported as “μmol DNPH/min.mg protein” but as “μmol DNPH/mg protein”. The authors state that the scientific conclusions are unaffected.

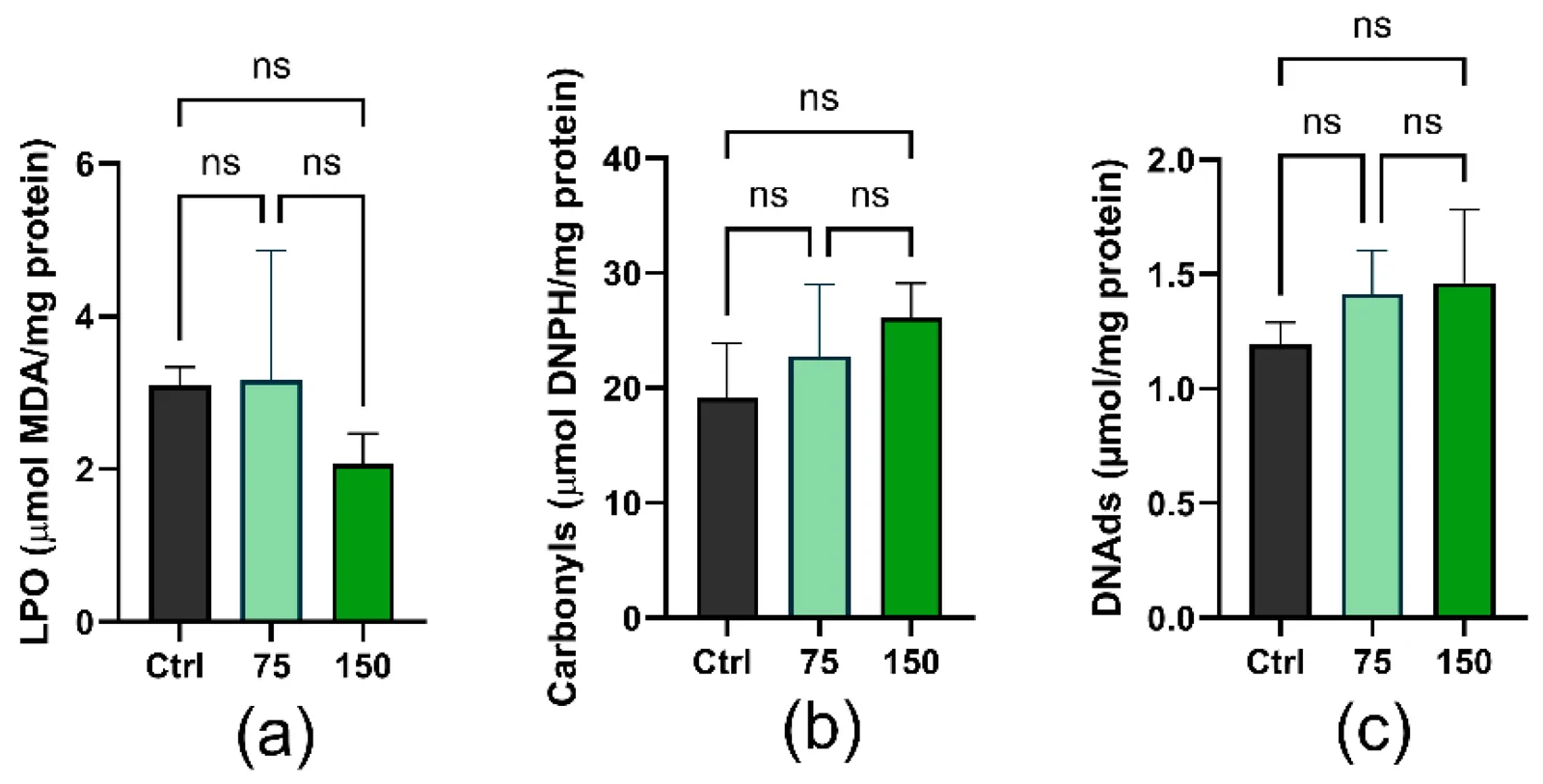



**Error in Figure 8**

In the original publication, there was a mistake in Figure 8 as published. Acetylcholinesterase (AChE) activity was incorrectly reported as “μmol NADH/mg protein” and should instead be expressed as “μmol TNB/mg protein”. The authors state that the scientific conclusions are unaffected.

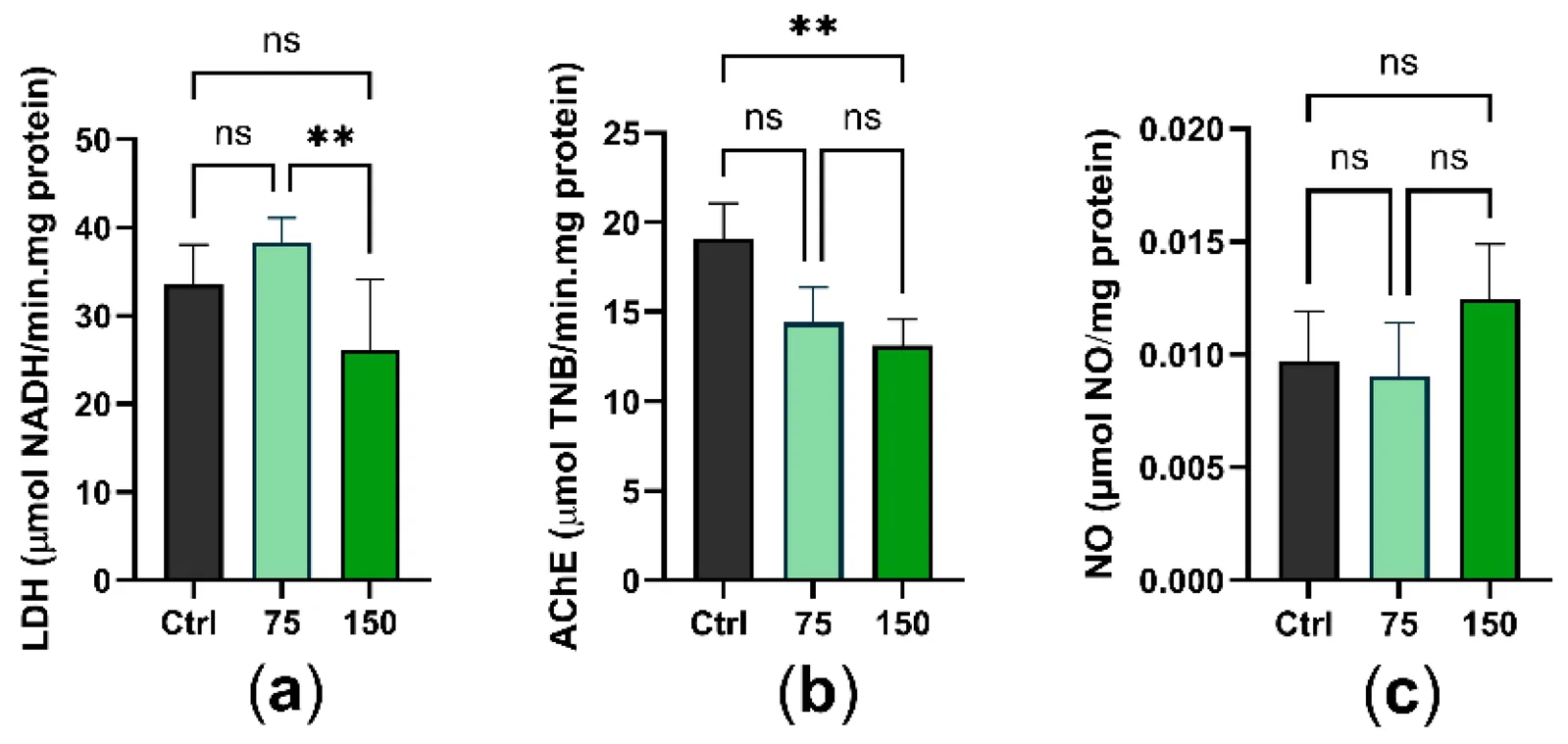



**Text Correction**

There was an error in the original publication. In the Methods section, glutathione S-transferase (GST) activity was incorrectly described as being calculated using the NADPH extinction coefficient. GST activity was in fact measured based on the formation of a conjugate between reduced glutathione (GSH) and 1,2-chloro-2,4-dinitrobenzene (CDNB) but using the CDNB extinction coefficient. Furthermore, ATPase activity was neither measured nor presented in the study and should not be mentioned.

A correction has been made to 4. Material and Methods, 4.7. Biochemical Analysis. The corrected text is below:

At 96 hpf, the surviving embryos from each replicate (around 30, n = 1) were collected and homogenized in cold HEPES buffer (0.32 mM sucrose, 20 mM HEPES, 1 mM MgCl_2_, and 0.5 mM phenylmethylsulfonyl fluoride [PMSF], pH 7.4) in a Biobase BHY-1 (Jinan, China). Following centrifugation at 12,000× *g* for 10 min at 4 °C, the protein of the supernatant was determined by measuring the absorbance at 280 nm, using a Take3 plate (BioTek Instruments, Winooski, VT, USA). The different enzymatic activities and oxidative stress markers were measured using either a PowerWave XS2 microplate scanning spectrophotometer (BioTek Instruments, Winooski, VT, USA) or a Cary Eclipse fluorescence spectrophotometer (Varian, Palo Alto, CA, USA) at 30 °C, as described before. The activity of superoxide dismutase (SOD) was evaluated according to its ability to inhibit the photochemical reduction of nitroblue tetrazolium (NBT) at 560 nm. Catalase (CAT) activity was determined by monitoring the reduction in absorbance of a hydrogen peroxide solution at 240 nm. The activities of glutathione reductase (GR) and glutathione peroxidase (GPx) were measured at 340 nm using the extinction coefficient of NADPH. Glutathione-S-transferase (GST) activity was quantified at 340 nm, based on the formation of a conjugate between reduced glutathione (GSH) and 1,2-chloro-2,4-dinitrobenzene (CDNB). The levels of GSH and oxidized glutathione (GSSG) were assessed with excitation at 320 nm and emission at 420 nm, and the GSH/GSSG ratio was used to calculate the oxidative stress index (OSI). Lipid peroxidation was measured at 530 nm via the reaction of malondialdehyde (MDA) with thiobarbituric acid (TBA). Protein carbonylation was determined at 450 nm, using the DNPH extinction coefficient. Lactate dehydrogenase (LDH) activity was assessed at 340 nm through the sodium pyruvate-mediated oxidation of NADH. Acetylcholinesterase (AChE) activity was quantified at 405 nm. DNA strand breaks were measured using excitation at 360 nm and emission at 450 nm [48].

The authors state that the scientific conclusions are unaffected. This correction was approved by the Academic Editor. The original publication has also been updated.
