# Supplementary material for: Effects of Eriobotrya japonica (Thunb.) Lindl. Leaf Extract on Zebrafish Embryogenesis, Behavior, and Biochemical Pathways
Source: Molecules. 2025 Aug 3;30(15):3252. doi: 10.3390/molecules30153252 (PMC12348488; doi:10.3390/molecules30153252)
Supplement: Supplementary file 1 [file molecules-30-03252-s001.zip › molecules-3759328-supplementary.pdf]

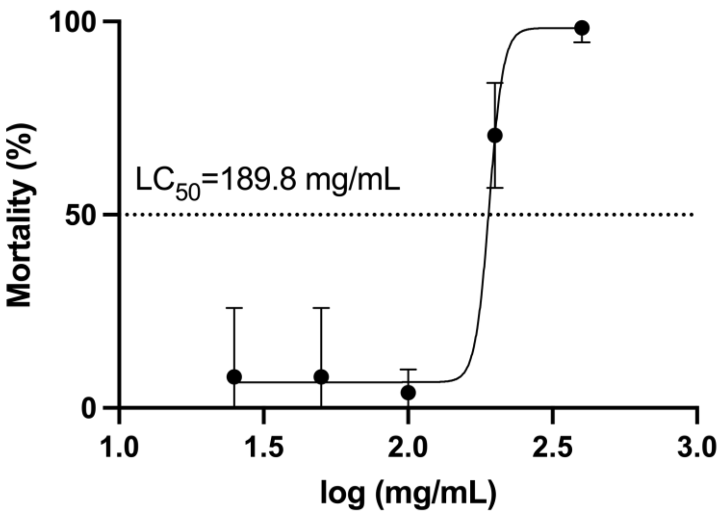

Figure S1. Concentration-response curve of zebrafish mortality following 96 h exposure to the extract of *Eriobotrya japonica* (loquat) leaves. Log-transformed concentrations are plotted on the x-axis as a function of the mortality, considering the control mortality. The 96-h lethal concentration (LC_{50}) value of the extract of *Eriobotrya japonica* (loquat) leaves was calculated as 189.8 mg/L
